# Microbiome stewardship: definition and guiding principles for implementation

**DOI:** 10.1093/sumbio/qvag028

**Published:** 2026-06-24

**Authors:** Kieran C O’Doherty, Mikaela Beijbom, Emma Allen-Vercoe, Mallory J Choudoir, Diego S Silva, Carla Bonilla, Justine Debelius, Sarah Elton, Aviâja Lyberth Hauptmann, Andreas Heyland, Nicolae Morar, Derek Skillings, Zhongzhi Sun, Patricia G Wolf, Robert G Beiko, Suzanne Lynn Ishaq

**Affiliations:** Department of Psychology, College of Social and Applied Human Sciences, University of Guelph, Guelph, ON N1G 2W1, Canada; Department of Psychology, College of Social and Applied Human Sciences, University of Guelph, Guelph, ON N1G 2W1, Canada; Department of Molecular and Cellular Biology, College of Biological Science, University of Guelph, Guelph, ON N1G 2W1, Canada; Plant and Microbial Ecology Department, College of Agriculture and Life Sciences, NC State University, Raleigh, NC 27695, United States; Faculty of Medicine and Health, University of Sydney School of Public Health, Camperdown, NSW 2006, Australia; Department of Biology, College of Arts and Sciences, University of San Diego, San Diego, CA 92110, United States; Department of Epidemiology, John Hopkins Bloomberg School of Public Health, Baltimore, MD 21205, United States; Social and Behavioural Health Sciences Division, Dalla Lana School of Public Health, University of Toronto, Toronto, ON M5T 3M7, Canada; Department of SILA, Institute of Health and Nature, Ilisimatusarfik - University of Greenland, 3905 Nuuk, Greenland; Department of Integrative Biology, College of Biological Science, University of Guelph, Guelph, ON N1G 2W1, Canada; Department of Philosophy and Environmental Studies Program, College of Arts and Sciences, University of Oregon, Eugene, OR 97403, United States; Department of Philosophy, College of Arts and Sciences, UNC Greensboro, Greensboro, NC 27412, United States; Faculty of Computer Science and Institute for Comparative Genomics, Dalhousie University, Halifax, NS B3H 1W5, Canada; Department of Nutrition Science, College of Health and Human Sciences, Purdue University, West Lafayette, IN 47907, United States; Faculty of Computer Science and Institute for Comparative Genomics, Dalhousie University, Halifax, NS B3H 1W5, Canada; School of Food and Agriculture, University of Maine, Orono, ME 04469, United States

**Keywords:** microbiomes, stewardship, public health, policy, ecosystem health, human microbiome, planetary health, One Health, health governance, ecosystem governance, ecological determinants of health

## Abstract

Microbiomes are essential for ecosystem, plant, animal, and human health. There is accumulating evidence that changes to microbiomes from anthropogenic activities are associated with adverse health outcomes. Despite this evidence and calls for action, almost no oversight mechanisms exist to protect microbiomes or their key functions, in part due to uncertainty about what to protect, and how. We have previously proposed microbiome stewardship as a foundational concept that can act across policy domains to facilitate the ongoing presence of key microbial communities and their functions. The purpose of this article is to provide a working definition of microbiome stewardship and develop guiding principles to support its implementation. The concept of microbiome stewardship is relevant to a wide range of policy domains, such as public health, clinical care, environmental protection, food production, and agriculture. Nonetheless, the implementation of microbiome stewardship will be highly specific, as it needs to be guided by considerations of microbial habitat, objectives of stewardship, and available opportunities for intervention. Accordingly, aligning stewardship responsibility with specific institutions and governance mechanisms will be context-dependent. We conclude with a discussion that situates microbiome stewardship relative to other initiatives.

**Sustainability Statement**

Microbiomes play an essential role in the health of humans, plants, animals, and ecosystems. The concept of microbiome stewardship can be applied across species, ecosystems, and policy domains to support health on local and global scales. This work supports progress predominantly toward the UN Sustainable Development Goal of One Health. It is also relevant to goals 3 (Good Health and Well-Being), 6 (Clean Water and Sanitation), 9 (Industry, Innovation, and Infrastructure), 11 (Sustainable Cities and Communities), 12 (Responsible Consumption and Production), 13 (Climate Action), 14 (Life Below Water), 15 (Life on Land), and 17 (Partnerships for the Goals).

## Introduction

In recent years, there has been increasing attention to concepts such as environmental health (Finn and O’Fallon 2015, World Health Organization 2025), One Health (World Health Organization 2022, Ginnan et al. 2025), and planetary health (Horton et al. 2014, Whitmee et al. 2015, Wabnitz et al. 2020). These approaches share a core idea: that the health of organisms is intricately connected with the health of other organisms and to the ecosystems in which they exist. A unifying mechanistic element of these interconnected systems are microbiomes: communities of microorganisms that live in, on, and around us. Microbial communities contribute essential ecological, metabolic, immune, and yet-unidentified functions to their hosts and larger ecosystems (Gilbert and Neufeld 2014). However, evidence is accumulating that many microbial ecosystems are changing as a result of human activities (Ishaq et al. 2019, Rocca et al. 2019, Groussin et al. 2021, Peixoto et al. 2024), and these changes are associated with adverse health effects for hosts and larger ecosystems (Athithan et al. 2026). In the case of humans, microbiome changes have been implicated in many conditions such as asthma (Kozik and Huang 2019, Barcik et al. 2020), lung infections (Yu et al. 2025), cancer (Hou et al. 2022), inflammatory bowel disease (Walters et al. 2014, Li et al. 2015, Holcomb et al. 2023), obesity (Borrego-Ruiz and Borrego 2025), and neuroinflammatory disorders (Bostick et al. 2022). Beyond human health, human activities have been associated with, for example, alterations to soil (de Vries et al. 2013, Tsiafouli et al. 2015), plant (Ishaq 2017, Trivedi et al. 2022, Shahid et al. 2024 ), and insect (Graber and Moreau 2026) microbiomes in ways that reduce chemical/nutrient cycling and availability, growth, and resilience to environmental stressors (Trivedi et al. 2022, Boyle et al. 2024).

Despite growing evidence for human-induced microbiome impacts, relatively little is being done to address these problems. Several approaches to safeguarding microbial life—discussed in more detail in the Discussion section—have gained attention in recent literature: a biobanking model (Dominguez Bello et al. 2025), a conservation model (Junker and Farwig 2025, Gilbert et al. 2025), and a rights-based model (Rizk et al. 2026). In addition, approaches such as One Health have brought awareness to the connectedness of the health of different organisms and have noted the important centralized role of microbes (Daisley et al. 2022, Ginnan et al. 2025). In parallel, some microbiome scientists have been strong advocates for recognizing the role of microbiomes in health and have been successful in promoting awareness of how individuals can improve the health of their individual microbiomes (Blaser 2011, Gilbert et al. 2018). While individual choices matter, systemic drivers—pollution, deforestation, and industrial food systems—can cause widespread microbiome alterations with harmful health consequences. These factors influencing microbiomes operate beyond individual choice or control, at the collective level (Ishaq et al. 2019 , Rocca et al. 2019, Elton 2021, Groussin et al. 2021). Yet the need to protect microbial life is not recognized or reflected in policy frameworks, regulations, or best practice guidelines across domains. Moreover, disruption and damage to microbiomes is experienced differently by different groups of people, which raises questions of equity and environmental justice (Choudoir and Eggleston 2022).

Previous discussions of microbiome stewardship (MiSt) (O’Doherty et al. 2014, Choudoir et al. 2025) have provided a rationale, but not yet a formal definition or pathways for implementation. The purpose of this paper is to develop the concept of microbiome stewardship as a foundation for guiding our relationships with microbial communities. We begin by developing a formal definition of microbiome stewardship. This is followed by considerations for enacting MiSt in practice and the need for governance mechanisms for monitoring and evaluation. We then discuss, from a microbiological perspective, which aspects of a microbiome should be stewarded based on current knowledge and available measurement metrics. Next, we propose a set of guiding principles for implementing microbiome stewardship. We conclude with a discussion of other approaches to safeguarding microbial life that we see as complementary to MiSt goals.

## Definition of microbiome stewardship

### Defining microbiomes

We define a microbiome as the collection of individual microorganisms and their genetic/genomic material within a habitat delineated by temporal, spatial, and environmental boundaries. We recognize this varies from other definitions proposed in the field (e.g. Marchesi and Ravel 2015, Tipton et al. 2019); however, we consider the abiotic factors and geophysical parameters a key component in stewardship, as the function of microorganisms cannot be divorced from their immediate environmental conditions. Thus, when we refer to microbiomes, we consider the individual microorganisms, specific functions, whole microbial communities, and overall ecosystem function to be entities which may require stewarding in different situations.

Microbiomes are shared across individuals (Turroni et al. 2017, Valles-Colomer et al. 2023) and species (Song et al. 2013), and host organisms source their microbiomes from their social and physical environments (Kembel et al. 2014, Trinh et al. 2018, Selway et al. 2020). Many of these microbial ecosystems are essential to the health and well-being of larger organisms in shared spaces. Thus, we need to understand microbiomes as *collective* phenomena rather than as attributes of individual organisms (like humans). Like air, water, and soil, microbial ecosystems are necessary for life on our planet. Microbiomes are therefore an important part of the biosphere and, accordingly, an ecological determinant of health (Elton 2021).

### Protecting microbiomes

There is a risk of degradation with all major systems of the biosphere, such as the air, water, and soils. This degradation has subsequent adverse effects for all who rely on these shared systems. When there is degradation of any parts of the biosphere’s systems, the effect is felt not only at the level of the individual, but at community, societal, and even global levels. Accordingly, addressing pollution of air, water, or soil is done most effectively as a collective. That is, the best solution to air pollution is not to profit by making clean air canisters for every individual, but rather to enforce regulation at a societal level that prohibits the release of pollutants that lead to low air quality. An example of collective action on a similar existential environmental issue is the Montreal Protocol, an international treaty signed in 1987 to phase out ozone-depleting substances. The Stockholm Convention on Persistent Organic Pollutants (Secretariat of the Stockholm Convention 2026) is another example of a global environmental treaty addressing very serious health problems that require large-scale collective responses.

We suggest a similar approach to microbiome stewardship, where collective action is taken to maintain the conditions needed to support robust microbial ecosystems that provide functions essential to host and ecosystem health. Individuals can and should make decisions that are beneficial for their microbiomes’ (and therefore their own) health, but such individual-level actions are insufficient for addressing larger systemic problems. Institutional, community, and international governmental regulations are needed across diverse domains to protect the conditions conducive to microbial biodiversity and the performance of key functions. The framework for MiSt we develop here is intended to support these collective efforts to sustain the microbiomes we rely on.

### Defining stewardship

The term stewardship has diverse meanings. Most generally, stewardship refers to taking care of something that has been entrusted to us or that we feel kinship towards (Saner and Wilson 2003, West et al. 2018). The notion of stewardship has been applied to many different things that have been deemed valuable and vulnerable, such as resources (e.g. crop seeds), ecosystems, data, antimicrobials, organizations, and cultural heritage. Many Indigenous cultures have long-standing histories of stewarding their environments. For example, the Māori people of Aotearoa (New Zealand) and First Nations communities in Canada have developed stewardship practices guided by complex and responsive understandings of their local ecologies. These practices have long been and continue to be driven by overarching values that prohibit the exploitation of, or disrespect to, other people, species, or places (Artelle et al. 2018). Stewardship may involve consultation, government oversight, voluntary industry actions, or frameworks that guide society toward resilience and well-being (Saner and Wilson 2003, Chapin III et al. 2015). There are two important questions when thinking about stewardship. First, what are the interests of the various affected parties? That is, who is affected by the issue and how? Second, who has the power, and therefore the responsibility, to do the stewarding? We return to these questions in the *Enacting MiSt* section of this paper.

### Defining Microbiome Stewardship (MiSt)

Given the diversity of meanings of stewardship, and the variability of microbiomes, it is important that we specify what we mean when we apply the notion of stewardship to microbiomes. We define microbiome stewardship as a set of evidence-based responsibilities and practices aimed at maintaining microbial biodiversity and microbiome functioning across microbial habitats, which in turn supports the health and well-being of humans, animals, plants, and ecosystems. MiSt entails conscious and strategic actions to conserve and protect microbiomes in the face of human and other activities that would lead to deteriorating health of humans, non-human life, and ecosystems. While recognizing that humans are just one species among all living beings on the planet, the responsibility lies with humans to desist from anthropogenic activties damaging microbial ecosystems. If beneficial relationships with microbiomes are threatened by human activities, then the onus is on humans to change our practices to be more life-sustaining. Effective microbiome stewardship requires collective sociopolitical action at all levels: institutional practices (e.g. hospital guidelines for prophylactic antibiotic use), national policies (e.g. regulation of agricultural pesticides known to harm microbial ecologies [Wan et al. 2025]), and global standards (e.g. international agreements on microplastics). Importantly, the actions taken at any level should not recreate practices of harm and inequity.

Because microbiomes vary widely across contexts, stewardship responsibilities must be assigned in a place–specific, ecologically situated, and context–dependent manner. Fundamentally, however, MiSt recognizes that (1) microbiomes are a dynamic and essential foundation for life on Earth and for the healthy functioning of host and environmental ecosystems; (2) microbiomes are collective phenomena, as different microbiomes are interdependent with each other, host ecosystems, and physical environments; and (3) action is needed to ensure the well-being of future generations by ensuring that they are born into environments that support microbial biodiversity and microbiome functions needed for health.

Applying the idea of stewardship can be complex even for more tangible substances or objects like water or heritage buildings. The nature of microbiomes and the varied roles they play in supporting the health of other host and environmental ecosystems makes applying the concept of stewardship to microbial ecologies even less straightforward. Microbes are generally seen to be the most resilient life forms on the planet. However, there are many kinds of microbes, and some are more resilient than others. Moreover, microbiomes can have high levels of functional redundancy; disparate taxa with different metabolisms and physiology can sometimes occupy similar ecological niches and carry out the same ecosystem process. Therefore, MiSt is not necessarily about the protection of all microbes or any specific microbe per se. While some stewardship efforts may target specific microbes, most aim to preserve key microbial functions, ecological networks, and host or ecosystem health. This is discussed in more detail elsewhere (Athithan et al. 2026).

Microbiomes are also highly dynamic and can change for many reasons (including human activity) on a continuous basis. Consequently, MiSt cannot be the static conservation of a particular state of a microbiome, and should not aim to leave microbiomes untouched, unchanged, or in an imagined “wild” state. Rather, MiSt employs intentional maintenance as needed, guided by careful assessment of potentially competing values and imperatives. Changes to microbiomes as the result of human actions are inevitable; however, the nature and consequences of these interactions are not. Nor are they easily predictable, especially when extrapolating to larger scales or complexity of systems.

Given all that remains unknown about microbiomes, successful microbiome stewardship also requires epistemic humility. The goal is to recognize knowledge gaps and maintain a critical, open-minded attitude that fosters intellectual integrity and supports sound decisions about safeguarding microbial life. It is certain that in years to come we will identify new microbiome functions and capabilities that are essential for a variety of hosts and ecosystems. Therefore, MiSt must support conditions that maintain known microbial functions while also preserving biodiversity to protect yet–unknown functions and the intrinsic value of microbes (West et al. 2018, Bossert and Höll 2025).

## Enacting microbiome stewardship across social domains

Effective implementation of stewardship requires specifying roles and responsibilities, and regulatory or other implementation mechanisms (Saner and Wilson 2003). A challenge specifically for the stewardship of microbiomes is that very different microbial ecological niches fulfill different kinds of essential functions, and in relation to diverse domains of human activities. Further, for the concept of MiSt to be robust and effective, it must be anchored in specific practices and policies, with clearly defined roles and locating of responsibilities. Therefore, stipulating what kinds of institutional and regulatory mechanisms are best for achieving stewardship goals, and where to assign roles and responsibilities, will depend on the issue and scope.

To make the abstract concept of microbiome stewrdship relevant, the nature of the problem(s) it should address needs to be clearly articulated. Key questions to ask in enacting MiSt are therefore: *What is the nature of the problem that MiSt is intended to address? What organisms or ecosystems are being threatened, and in what way are microbiomes implicated?* For example:

- To help address rising IBD rates linked to harmful food additives (Walters et al. 2014, Li et al. 2015, Chassaing et al. 2022), stewardship may involve regulating these specific substances.
- In response to the widespread loss of bee colonies, which has been associated with agrochemicals and subsequently altered bee microbiomes (Daisley et al. 2022), MiSt might focus on regulatory solutions to manage pesticides in specific geographic locations.
- Microbes have an important symbiotic role in coral functioning (Sweet et al. 2021). The accelerated loss of coral reefs is a result of broad environmental stressors, but preserving the microbial symbionts may be an important mitigation strategy. In this case, MiSt might transcend regulatory and geographic demarcation to facilitate international cooperation.

Enacting stewardship will differ dramatically in each of these examples because the problems are embedded in different social systems and institutions, not to mention different regulatory systems across jurisdictions. In many instances, MiSt actions might be most effective when integrated with existing initiatives. For example:

- Overuse of antibiotics in livestock is a major concern as it contributes to the emergence of antibiotic resistance (Silvestro et al. 2025). Overuse of antibiotics also induces changes to microbiomes that are associated with adverse health effects for animals and humans, thus making it an important area of intervention for MiSt. In this instance, MiSt can act in tandem with antimicrobial stewardship and support existing AMR and One Health initiatives.
- Increasing microplastics pollution in aquatic environments affects microbial ecosystems and biogeochemical processes in ways shown to contribute to climate change (Chen et al. 2022, Sunil et al. 2024). Various policy measures to address these threats have been adopted across countries, but with disparities in scope, implementation, and enforcement (Bhatt et al. 2026). MiSt considerations can support these efforts.
- Air pollution has been shown to affect the composition and function of the gut and lung microbiomes, alterations that are associated with changes in asthma risk (Howard et al. 2019). Air pollution is largely recognized as a significant health threat, and MiSt is aligned with and can support policies and investments geared towards reducing emissions around the world through cleaner transport, energy, power generation, waste management, and more.

### Roles and responsibilities

Activities relating to MiSt need to be assigned to specific persons or institutions. Responsibility for stewardship practices can be located in a range of places, including governments (at national and subnational levels), industry leaders and organizations, NGOs, local community groups, and partnerships between governments, industry, and community members. Enacting microbiome stewardship therefore involves questions about which persons or institutions should be responsible and empowered to implement MiSt activities in any given context. In some instances, new institutions (i.e. an independent governing board) may need to be created that bestow responsibility and power to enact specific stewardship activities.

Consideration also needs to be given to individuals and communities who are impacted, knowingly or not, by societal practices that have adverse effects on microbiomes. *What groups have an interest in (i.e. are affected by) these health issues? What are their roles and responsibilities in relation to other communities and larger society?* People most affected may lack decision–making power, but their lived experience is essential for designing effective initiatives and implementing stewardship practices. For example, physicians are important people to consult and involve when designing regulations about antibiotic prescribing. Similarly, patients and parents may not have the power to determine regulations and guidelines, but they will have very important insights about patient needs, everyday practices, and more.

The effective allocation of roles and responsibilities also requires identification of relevant expertise. The complex, interdisciplinary, and diverse landscape of microbiome stewardship implies that its implementation requires very diverse sets of expertise and collaborative efforts. For example, identifying suitable metrics for key functions of the gut microbiome to guard against *Clostridioides difficile* infection requires very different expertise from engaging municipal governments to enact policies that make dumping of certain pollutants in local rivers and lakes illegal.

### Implementation mechanisms

Depending on the jurisdiction, the kinds of microbiomes being considered, and which health or environmental issues are being tackled, different mechanisms for societal decision-making may be available. Although MiSt may be anchored in personal commitments and values, it will ultimately require mechanisms for societal decision-making, including laws, policies, or regulations instituted in relevant domains (e.g. food, health, manufacturing, etc.), codes of ethics, professional standards (e.g. for doctors, foresters, farmers, etc.), institutional best practices (e.g. in agriculture, hospitals, etc.), and cultural and social expectations. Different mechanisms will operate on different levels of society (e.g. local community level, institutional, national, and global), and the most appropriate and effective level to target will depend on the issues of concern. Enacting MiSt thus requires addressing questions about *what societal or community mechanisms are available to address the specific problems being considered*.

In some instances, MiSt goals may be in alignment with existing initiatives. If that is the case, it may be possible to integrate considerations of microbiomes in existing policy and regulatory frameworks. For example, MiSt guidelines for prophylactic antibiotic use will also be of interest for existing antimicrobial stewardship initiatives. In other cases, concerns raised by altered microbiomes may be completely outside the scope of any existing governance mechanisms. In such cases, it may be necessary to develop novel implementation mechanisms. Integrating MiSt concepts into science education through place–based and critical pedagogy can support long–term, society–wide affectual shifts toward planetary health (Gruenewald 2003).

## The objects of stewardship

Microbiomes can be assessed, described, and summarized in many ways, but the ideal representation is not obvious in many cases (Roswell et al. 2021). What, precisely, is it that should be stewarded? Is it specific strains of microbes that directly contribute key functions to a system? Should entire microbial communities be considered to preserve ecological interactions that may support those key strains? Or should we focus on the metabolic functions that a microbial community confers upon its host and ensure that this is maintained? The answers to these questions will be system specific, and some are discussed in greater detail elsewhere (see Athithan et al. (2026) for a conceptual framework for stewardship at different scales, from host niche to ecosystem level, and examples of challenges and opportunities for policies at each scale.)

Taxonomic descriptions are valuable in assessing the similarity of sampled communities, and the presence of specific taxa may be broadly interpreted as indicators of functions and ecological roles. However, not all members of a taxon contribute equally to the desired function. For example, *Candidatus* “Accumulibacter phosphatis" sub-clades can differ in their contribution to enhanced biological phosphorus removal in wastewater treatment (Kolakovic et al. 2021); vaginal *Lactobacillus* species such as *L. crispatus* and *L. iners* differ in genomic features and protective potential for vaginal health (France et al. 2016); and members of the Lachnospiraceae family vary in genomic and functional capacities related to short-chain fatty acid and butyrate production (Sorbara et al. 2020). In some cases, community states can be inferred from indicator taxa, such as *Akkermansia muciniphila* for gut health or *Fusobacterium* spp. for adverse outcomes (Wang and Fang 2023, Ioannou et al. 2024). Still, linking a taxon to microbiome function assumes all its members perform the same role and that no other taxa can substitute—assumptions that often fail, because strains within a species differ widely in gene content and functional potential (Xiaomei M et al. 2023, Yang et al. 2023), and because functions such as mucin glycan degradation and antibiotic resistance are widespread across many phylogenetically divergent lineages (Glover et al. 2022, Wang et al. 2026). These assumptions are invalid for many taxa in many microbiomes in a multitude of habitats: even within the same species, strains can differ substantially in gene content, with a shared core genome but large, strain-specific accessory gene repertoires, leading to meaningful variation in functional potential (Shoer et al. 2024). Functional redundancy allows different taxa to perform similar roles, making community function more stable despite taxonomic turnover (Eng and Borenstein 2018, Tian et al. 2020).

An alternative to targeting specific taxa is to consider the total diversity of a microbiome. In many settings including the human gut, a more diverse microbiome (however defined) is often seen as indicative of a robust and resilient community that can buffer disruptions such as introduced toxins and invasive pathogens (Spragge et al. 2023, Van Hul et al. 2024). In contrast, in *Lactobacillus*-dominant human vaginal communities, lower diversity is often associated with a healthy vaginal state (Ravel et al. 2010, Ma et al. 2012). A diversity-centric approach, which would align also with the goals of the microbial conservation model, can reduce the need to define and assess individual taxa, although the choice of taxonomic rank may aggregate or separate critical groups of microbes (Debelius et al. 2020, Scholss 2021, Armour et al. 2022). The correct diversity measurement is likely tied to the type of perturbation being explored. Temporal community dynamics may also be important, requiring sampling at key time intervals to assess the stability and state of a system (Halfvarson et al. 2017, Zaneveld et al. 2017).

However, if our main motivation for stewardship is to preserve specific metabolic functions or ecological roles, why not steward these functions directly instead of trying to find the right taxonomic package to protect or reconstruct? Many organisms across the tree of microbial life can fix nitrogen, or synthesize butyrate, or assemble key secondary metabolites of vital importance to macroorganisms. In such cases, it may be enough to retain any taxon or taxa with the desired capabilities, regardless of their specific identity. But a strict focus on function may overlook vital context that determines the viability and utility of a microbiome. Nitrogen fixation will be of little use if a given organism cannot associate with key plant species or survive at a given soil acidity; butyrate synthesis will fail in the absence of key precursor compounds and regulatory cues; and secondary metabolite pathways may be unreliable if they are housed on mobile genetic elements that are transient in a population. It is difficult to conceive of stewardship without considering functions and roles, and there will be many cases where a focus on function alone will not be sufficient.

Practical considerations will also play a key role in what we steward. Some interventions, such as direct introduction of specific microbes or the use of compounds that promote their growth (i.e. probiotics and prebiotics in humans, and analogues such as bioremediation in other environments), are feasible if conditions support their persistence. Other cases, such as those that introduce complex management practices, may be limited to more general promotion of microbial diversity, resilience, and stability instead of promoting very specific taxa or functions.

## Guiding principles of microbiome stewardship

To support our definition of microbiome stewardship, we propose a series of guiding principles. We intend for MiSt to serve as a unifying concept that can be applied across diverse domains of human activity and contexts (conservation, human health, animal health, agriculture, etc.) to emphasize the importance of safeguarding microbiomes for human, animal, plant, and ecosystem health (Choudoir et al. 2025). The guiding principles are therefore intentionally broad to allow them to be applied across different contexts and are based on the definition and discussion we have provided above. We give examples of how different applications of principles might look in practice.

**Figure 1:**
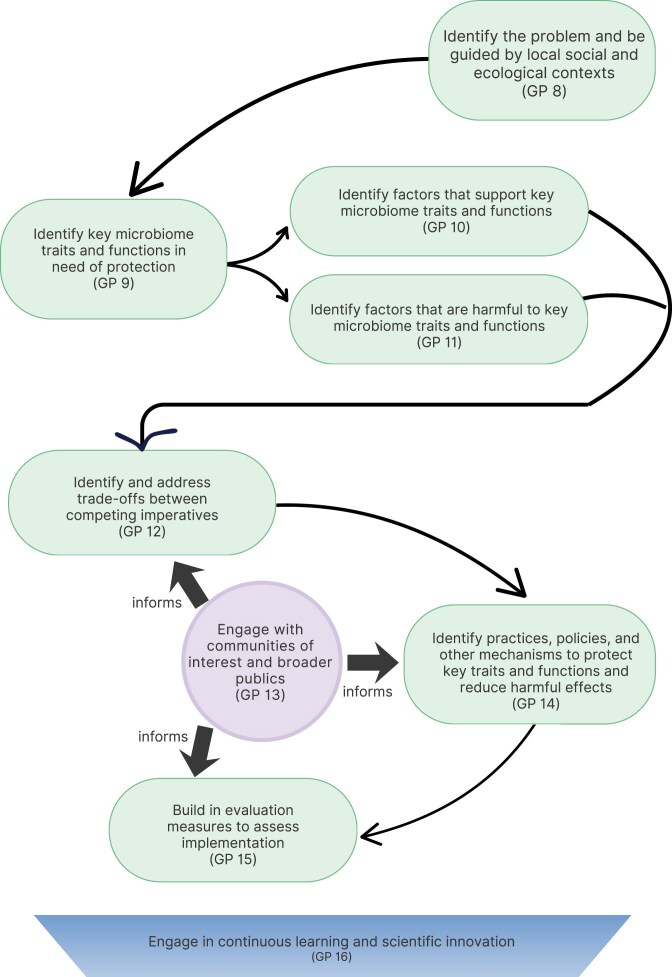
MiSt actions flowchart.

We divide the principles of MiSt into three sections. The first, *Premises*, includes principles that have to do with the knowledge and awareness that are necessary as a foundation for MiSt. The second section, *Responsibilities*, includes principles that have to do with recognizing imperatives that follow from acceptance of the premises. The third section, *Actions*, includes principles that stipulate how the responsibilities of MiSt can be fulfilled (see Table 1 for an overview of all guiding principles; see Fig. 1 for a flowchart of the MiSt Action guiding principles).

**Table 1:** Guiding principles overview.

Principle type	Guiding principle
**Premises** *Knowledge and awareness that are necessary as a*	1. Recognition that we are dependent on microbiomes to fulfill essential functions in larger ecosystems and in maintaining the health of humans, animals, and plants
*foundation for microbiomestewardship*	2. Recognition that microbiomes are collective ecological determinants of health
	3. Recognition that human activities are causing changes to microbiomes resulting in adverse effects for human, animal, plant, and ecosystem health
	4. Recognition that differential exposures to changing microbial communities are causing unequal, differentiated harms to different populations, often compounding existing social inequalities
**Responsibilities** *Imperatives and values that follow from acceptance*	5. Modify societal practices to mitigate, prevent, or reverse changes to microbiomes associated with adverse effects for hosts or ecosystems
*of the premises*	6. Respect and care for future generations
	7. Respect resource and data sovereignty, and do not perpetuate legacies of resource extraction
**Actions** *How the responsibilities of microbiome stewardship*	8. Identify the specific problems microbiome stewardship practices are intended to address, guided by local social and ecological contexts
*can be fulfilled*	9. Identify key microbiome traits and functions that are important and in need of protection for supporting host and ecosystem health in specific contexts and set these as targets for stewardship practices
	10. Identify environmental factors that are essential to and supportive of key microbiome traits and functions, as well as to microbial biodiversity, and ensure the continued presence of these factors
	11. Identify factors that are harmful to key microbiome traits and functions, and to microbial biodiversity
	12. Identify and address trade-offs between competing imperatives and involve relevant actors and affected individuals and groups when doing so
	13. Engage with communities of interest and broader publics to ensure that microbiome stewardship activities are informed by local knowledge and guided by the aims and concerns of those most affected by the issues under consideration
	14. Identify practices, policies, and other social and institutional mechanisms to (a) protect key microbiome traits and functions and (b) eliminate/reduce harmful effects
	15. Build in evaluation measures to assess successes and failures of stewardship practices
	16. Engage in continuous learning and scientific innovation

### Premises

#### Recognition that we are dependent on microbiomes to fulfill essential functions in larger ecosystems and in maintaining the health of humans, animals, and plants

Microorganisms provide sustenance for organisms including protozoa and zooplankton, filter-feeding animals, insects, and, in turn, larger organisms, including plant and animal life (Gilbert and Neufeld 2014). They provide essential metabolic functions to other organisms and to entire ecosystems (Gilbert and Neufeld 2014, Ishaq et al. 2019, Peixoto et al. 2024, Anthithan et al. 2026). Humans depend on microbial ecosystems for development, immunity, and metabolic activities, and research is increasingly focused on the role of the microbiome as a modulator of myriad health and disease states (The Human Microbiome Project Consortium 2012, The Integrative HMP Research Network Consortium 2019, Lebeer et al. 2023). In animals, microbiomes are critical for immune system maturation, digestion of plant fibers and beneficial microbial byproducts, reproductive fitness, and nearly all facets of health (Chung et al. 2012, Williams et al. 2018). In plants, host microbiota can influence growth and survival from environmental stress (Ishaq 2017, Trivedi et al. 2022, Shahid et al. 2024). At the planetary level, diverse environmental microbiomes are involved in the regulation of the planetary climate, governing the exchange of carbon and nitrogen between the atmosphere and terrestrial biosphere (Cavicchioli et al. 2019). These insights are also aligned with One Health approaches.

As the importance of microbiomes for all life forms is increasingly explored and understood, MiSt involves recognizing that humans, animals, and plants could not lead healthy lives without microbiomes performing key life-sustaining functions.

#### Recognition that microbiomes are a collective ecological determinant of health

Microbiomes are shared across individuals and species, and microbiomes that are associated with particular host organisms are sourced from their environments (i.e. horizontal inheritance) or through vertical inheritance (Skillings 2016, Turroni et al. 2017, Kaur et al. 2021, Choudoir and Eggleston 2022, Valles-Colomer et al. 2023). Individuals can only source microbes that are present in their environments. Thus, the ability to encounter, acquire, and maintain microbiota is subject to the same ecological dynamics which affect the microbial communities in a given environment. Environmental stressors have the potential to affect the health of entire communities, not just individuals (Rocca et al. 2019). Therefore, to optimize health of humans and non-humans, microbiomes need to be understood as shared ecological determinants of health (Ishaq et al. 2019, Elton 2021, Rook 2022) that need to be safeguarded at the collective level, in the same way that air, water, and soil are.

#### Recognition that human activities are causing changes to microbiomes resulting in adverse effects for human, animal, plant, and ecosystem health

Across species and ecosystems, accumulating evidence suggests detrimental changes to life-supporting microbiomes due to industrialization (Sonnenburg and Sonnenburg 2019, Rappuoli et al. 2023). For example, excessive synthetic fertilization can alter soil bacterial nitrogen cycling and lead to the accumulation of toxic nitrogen products and increased greenhouse gas emissions (Carr et al. 2025). Air pollution changes activity in and degrades the gut microbiome, leading to inflammatory and metabolic disorders (Salim et al. 2013). Pesticides negatively alter bee microbiomes (Daisley et al. 2022). Agricultural and land use intensification reduces soil biodiversity (de Vries et al. 2013, Tsiafouli et al. 2015), which is linked to reduced ecosystem functionality (Wagg et al. 2014). Drugs and other xenobiotics can profoundly and negatively affect human and animal gut microbiome composition and function (Spanogiannopoulous et al. 2016, Gerasimidis et al. 2019, Lindell et al. 2022).

Microbiomes are dynamic yet not invulnerable. Plasticity and self–adjustment have limits, especially under rapid, large–scale, or novel anthropogenic pressures; therefore, safeguarding the conditions that sustain key microbial functions remains necessary. Stewardship requires recognizing that human actions harm microbiomes and that humans also have the power to change these actions.

#### Recognition that differential exposures to changing microbial communities are causing unequal, differentiated harms to different populations, often compounding existing social inequalities

Specifically in the context of human microbiomes, microbial exposures and our reactions to them are not homogeneous. Human-constructed social categories, systems, and systemic and institutional discrimination affect access and exposure to microbiomes, with corresponding health impacts (Rook 2022). For example, gendered marketing based on social expectations affects lifestyle and behaviors in ways that can shape microbial exposures and microbe-host interactions, ultimately impacting host health (Jenkins et al. 2018, Bärebring et al. 2020, Devries and Jakobi 2021, Eriksson et al. 2022). In many countries, there is a lack of policies regarding parental support and breastfeeding practices for establishment of early life microbiomes (Wicklund et al. 2024). While “race” is not a meaningful biological categorization for humans, the social and ideological processes of racialization and their material consequences can have significant effects on microbiomes and health (Benezra 2020, De Wolfe et al. 2021).

Built environments also differentially shape microbial exposure. For instance, green and blue spaces can provide beneficial microbial exposures. Access to these is often lower in older and low–income neighborhoods, disproportionately affecting Indigenous and racialized communities (Ishaq et al. 2019, Warbrick et al. 2023). Furthermore, industrial food systems not only contribute to the degradation of microbiomes via intensive and high-disturbance farming practices, but also produce significant amounts of ultra-processed foods that have deleterious effects on microbiomes that have beneficial functions for health (Hauptmann et al. 2020, Wolf et al. 2022). Social forces are therefore deeply integrated into human biology through microbial mechanisms. MiSt involves recognizing the social forces that differentially shape microbial exposures and corresponding harms and intervening where possible to minimize such inequities.

### Responsibilities

#### Modify societal practices to mitigate, prevent, or reverse changes to microbiomes associated with adverse effects for hosts or ecosystems

Given the importance of microbiomes for health, it is essential that we formally recognize a responsibility to ensure the maintenance of key microbiome functions and microbiomes overall (Gilbert et al. 2025) as a foundation for specific actions that support microbiomes and their functioning. It is important to recognize that detrimental human-induced changes to microbiomes are not inevitable. Factors that cause these changes should be eliminated or their negative effects on health mitigated by institutional, national, and global mechanisms. When negative health effects are associated with social inequalities, additional efforts should be made to mitigate these inequalities.

#### Respect and care for future generations

Our individual and collective actions and institutionalized practices have consequences for future generations. There is a collective responsibility to ensure that today’s decisions are sustainable for long-term microbial, human, plant, animal, and ecosystem health. Meeting the needs of the present should not compromise the ability of future generations to meet their own needs (United Nations 1987). We have a responsibility to ensure that there is a life-sustaining and healthy place for humans and non-humans to be born far into the future. This is also emphasized in Indigenous stewardship approaches, One Health, planetary health, conservation approaches, and by the Microbiota Vault. MiSt entails a responsibility to ensure that our interactions with, and impacts on, microbiomes are such that key microbiome functions and microbial biodiversity persist for future generations.

#### Respect resource and data sovereignty, and do not perpetuate legacies of resource extraction

The recognition that the microbiomes of industrialized societies seem to be changing in a manner associated with adverse health effects has led some to study the microbiomes of Indigenous communities around the globe. Unfortunately, many such research relationships have tended to be exploitative with benefits to researchers and members of industrialized societies, and no benefits to, or even harms to, the Indigenous communities (Mangola et al. 2022, Jones 2024). It is imperative that microbiome stewardship practices are carried out in partnerships with Indigenous and other communities with permission and in non-extractive ways. Community-based participatory research and community-inclusive approaches for research conducted with local communities, including Indigenous communities, should guide the development of MiSt initiatives. MiSt must follow ethical practices (Good et al. 2026). For instance, microbes collected from Indigenous communities must not be treated as a resource for the benefit of predominantly non-Indigenous industrialized nations (Redvers et al. 2020, Bader et al. 2023, Handsley-Davis et al. 2023, Warbrick et al. 2023).

### Actions

#### Identify the specific problems microbiome stewardship practices are intended to address, guided by local social and ecological contexts

The concept of MiSt is intended to be broadly applicable. However, specific stewardship practices need to be targeted. An essential step in implementing microbiome stewardship is identifying the problem or set of issues that it is supposed to address in a particular situation.

Implementation of MiSt and evaluation of its effectiveness also need to be guided by local social contexts and informed by local ecologies (i.e. the complex relationship between organisms and their environment in a locally defined geographic area). Microbiomes are locally adapted and have specific and situated roles and impacts (Danko et al. 2021, Lin et al. 2022, Ruff et al. 2024). Biological and social contexts are two different but crucially important aspects of how microbiomes are situated. Biologically, criteria for defining the key functions of any given microbiome must take into consideration the ecological, spatial, and temporal habitat designations, and the functional role that a microbiome fulfills for host and environmental ecosystems. Socially, the cultural, political, and institutional contexts which influence microbial exposures in unique ways must also be accounted for when considering relevant mechanisms for implementing and evaluating MiSt. Examples of such considerations include food systems, built environments, public health regulations, and employment norms and policies in which microbiomes are situated.

Thus, effective microbiome stewardship requires clearly defining the issues to address, understanding the situated nature and impacts of microbiomes, and identifying strategies to ensure the continuation of essential functions.

#### Identify key microbiome traits and functions that are important and in need of protection for supporting host and ecosystem health in specific contexts and set these as targets for stewardship practices

A next step is to identify the attributes of microbiomes that need to be stewarded. In the context of the health problems identified, which microbiomes or which *attributes* of a *specific microbiome* need attention? Targets for stewardship practice need to be quantifiable (or at minimum qualifiable). Importantly, microbiome sampling, characterization, and interpretation methods need to be accessible (within domain or area of expertise). Measured traits need to be predictive of a beneficial or detrimental health or environmental outcome, and the strength of the prediction must be accepted by the field in question (e.g. guided by public health targets or environmental quality standards) (O’Malley and Skillings 2018).

#### Identify environmental factors that are essential to and supportive of key microbiome traits and functions, as well as to microbial biodiversity, and ensure the continued presence of these factors

Once the microbiomes to be stewarded have been identified, it is necessary to identify the factors that support the existence and functioning of those microbiomes. Hosts recruit microbiota to their ecosystem via diet or metabolites (Yang et al. 2025) and exploring host biology can elucidate these factors. However, consideration of host-microbe-environment interactions requires knowledge of all three: for example, environmental exposures affect the establishment of biofilms on coral reefs, and those biofilms affect the ability of coral larvae to settle or not (Dittami et al. 2021, Saha et al. 2024, O’Brien et al. 2025).

In addition to recognizing the biotic and abiotic dimensions of life-supporting microbial habitats, stewardship should also consider landscape–level microbial habitats that maintain diversity and help reseed ecosystems after disturbances. The demographics of microbial populations, as well as ecological processes (such as chemical cycling and energy transfer) inform the stability of the members and functions in a microbiome and their resilience to disturbance (Shade et al. 2012, Shade 2023). Yet, without sufficient environmental support, no microbial community can be maintained indefinitely. For example, without sufficient green space and macro-biodiversity in urban environments to harbor microbiota, no amount of built infrastructure engineering will sufficiently replace environmental microbial exposures. In ensuring the presence of necessary environmental factors to support key microbiomes, we also need to acknowledge limitations on our ability to understand, let alone control, ecosystem processes.

#### Identify factors that are harmful to key microbiome traits and functions, and to microbial biodiversity

When changes to microbiomes have adverse effects on hosts and ecosystems, it is important to identify the factors that lead to these changes. After identifying target microbiomes and supportive conditions, the factors that lead to loss of key attributes of the microbiome must be identified (e.g. environmental toxins, food additives, and features of the built environment). MiSt involves identification of the specific factors that are involved in altering microbiomes to prevent, mitigate, or reverse ill health and ecosystem damage. For example, the use of pesticides on agricultural soils detrimentally affects non-target micro- and macro-organisms, which reduces the functionality and nutritional content of soil over time (Wan et al. 2025), which in turn adversely affects crop productivity (Swaine et al. 2025). Similarly, the addition of emulsifiers, including carboxymethylcellulose and sucrose fatty acid esters to food negatively affect gut microbiomes with adverse implications for immunological development and function (Panyod et al. 2024).

Identifying the harmful impacts of such activities is an important step in microbiome stewardship. Ideally, efforts should be dedicated to stopping the use of harmful chemicals or practices in the first place. Where possible, action to reverse detrimental impacts should be taken. At minimum, efforts should be invested into identifying ways to mitigate harmful impacts.

#### Identify and address trade-offs between competing imperatives and involve relevant actors and affected individuals and groups when doing so

Trade-offs and competing imperatives will inevitably be encountered when engaging in MiSt. These considerations may be at biological, social, or economic levels, and must be identified, made explicit, and navigated in a participatory manner.

For example, biodegradable plastics were developed as promising alternatives to petroleum-based plastics because they do not produce persistent microplastics. However, recent research on polylactic acid (PLA), an emerging biodegradable plastic, has shown effects on bacterial diversity, composition, and function in estuarine sediments (Chen et al. 2022), as well as on soil microbial communities (Yi et al. 2024). Evidently, actions that appear to provide benefit to some host, ecosystem, or microbiomes, might lead to changes associated with adverse effects in other microbial communities and could have distinct effects across species and ecosystems (e.g. benefits to some, but harm to others). Similarly, green wall infrastructure that would have beneficial effects on important microbiomes would require higher humidity levels than many homes currently have. This may be in tension with HVAC systems of built spaces that are currently engineered to limit mold and regulate for a specific humidity.

Because microbiomes are complex and only partially understood, stewardship decisions must acknowledge uncertainty and the possibility of unintended consequences. Trade-offs should therefore be evaluated not only in biological or economic terms but also through an equity lens, recognizing that harms and benefits may be distributed unevenly across communities. These considerations reinforce the core rationale of MiSt: to act responsibly despite uncertainty while ensuring that stewardship choices do not amplify existing social or ecological inequities.

While some trade-offs can be anticipated, others might only become apparent in the ideation or implementation phases of a MiSt initiative. MiSt will require careful, conscientious attention to trade-offs and competing interests and their consideration in stewardship activities.

#### Engage with communities of interest and broader publics to ensure that microbiome stewardship activities are informed by local knowledge and guided by the aims and concerns of those most affected by the issues under consideration

Participatory principles should be adopted in MiSt efforts to ensure that local interests and concerns inform the navigation of trade-offs, competing interests, and MiSt activities. Because microbiomes are shared, and decisions and actions that occur at societal and institutional levels impact collective microbiomes, MiSt decision-making and initiative development should be informed by individual and community inputs (O’Doherty et al. 2013, Mangola et al. 2022, Jones 2024).

Impacted publics should be engaged in advancing an understanding of the nature of the problems they face regarding damaging microbiome changes and in developing solutions to these challenges. It is imperative to consider who is affected, which communities are impacted, how they are included in decision-making, and whose interests are being served by different approaches. The perspectives and interests of the communities affected by a given microbiome-related challenge or intervention should be actively solicited, carefully considered, and inform the design of contextually appropriate solutions and the best mechanisms for their implementation.

#### Identify practices, policies, and other social and institutional mechanisms to (a) protect key microbiome traits and functions, and (b) eliminate/reduce harmful effects

Once specific factors are identified that support or threaten key microbiome traits or functions, it is important to identify suitable interventions. These may involve changing institutional practices, national policies or laws, or international agreements. For example:

- changing the composition of cleaning products to remove unnecessary antimicrobials; guidelines for prophylactic antibiotic use; cooperation with governments to increase diverse food access
- regulation of agricultural pesticides known to harm essential microbial ecologies; parental leave that supports breastfeeding; adding microbiology to elementary and secondary curricula; zoning regulations that consider microbial ecosystems
- international agreements on persistent organic pollutants.

In developing such interventions, differential exposures across communities should be considered. This step of microbiome stewardship should therefore be informed by meaningful public and community engagement (see GP 4 and 13, above).

Ongoing vigilance and integration into larger policy structures will be required for the success of these interventions. Therefore, governance structures need to be established that represent the mandate of ongoing maintenance of environments that foster microbial biodiversity and microbiome key functions and the health of all living things that depend on them. The purpose of such governance structures would be to set standards for MiSt, monitor microbiome changes and functions, and enforce policies and practices safeguarding microbiomes. MiSt governance should be informed by the rest of the guiding principles discussed.

#### Build in evaluation measures to assess successes and failures of stewardship practices

Any stewardship interventions must be monitored to determine whether they work and to detect unintended consequences. For these reasons, it is important that evaluation frameworks are built into MiSt initiatives. These evaluations will involve metrics to assess outcomes (see Objects of Stewardship, above). Importantly, evaluations will have to be tailored to each initiative, its relevant contexts, and implicated local ecologies. Tailored evaluations will also support the identification of unintended effects or a lack of results, which will signal the need for deeper investigation and prompt adaptation.

MiSt involves building tailored evaluation plans into initiatives to enable researchers, practitioners, and policymakers to understand and communicate their impact.

#### Engage in continuous learning and scientific innovation

Although microbiome science has made rapid and impressive advances in the last 20 years, there is far more we do not know about microbiomes and their relationship to the health of other organisms and ecosystems. Importantly, we still know only very little about the long-term consequences of human activities on microbiomes, especially those relating to the creation of novel compounds and their release into environments (and host environments including humans).

For instance, glyphosate, a manufactured compound, is an active ingredient in many herbicides and is a major agricultural chemical in many countries, including Canada, the USA, and throughout the European Union (Health Canada 2017, United States EPA 2024, European Commission, n.d.-b). Recent studies link this compound to reduced microbiome diversity, loss of anti-inflammatory taxa like *Lactobacillus* and *Bifidobacterium*, and increased levels of potentially harmful bacteria (Tang et al. 2020, Liu et al. 2022, Lehman et al. 2023). When considered through the lens of MiSt, the findings of these studies warrant further research, a re-evaluation, and potential adjustments to the regulation and use of these compounds.

What is thought to be conducive to long-term sustainability at one time might later be shown to be detrimental. This does not mean that we should not act. Rather, it highlights the importance of continued pursuit of knowledge and progress monitoring, a commitment to being responsive to new information, and the importance of innovative sampling approaches and methods for monitoring microbial ecologies and health outcomes. Different methods can be used to monitor and characterize the microbiome, including amplicon sequencing for taxonomic profiling and shotgun metagenomics for assessing both the taxonomic composition and the functional potential of microbial communities. Metatranscriptomics, metaproteomics, and metabolomics can further provide insights into the active or realized functionality of the microbiome. The choice among these methods should depend not only on the specific questions being asked, but also on practical considerations such as cost-effectiveness, scalability, and feasibility for use in larger populations.

## Discussion and conclusion

We have presented a working definition for microbiome stewardship, considerations for its enactment, potential microbial objects to be stewarded, and guiding principles for implementation. As we noted in our introduction, the central idea that microbiomes are at risk and that a suitable response is required has been recognized by others. Several approaches to safeguarding microbial life have gained attention in recent literature: a biobanking model (Dominguez Bello et al. 2025), a conservation model (Gilbert et al. 2025, Junker and Farwig 2025), and a rights-based model (Rizk et al. 2026). Approaches such as One Health have also brought awareness to the connectedness of the health of different organisms and have noted the important role of microbes (Rappuoli et al. 2023, Ginnan et al. 2025). It is important, therefore, to situate the concept of MiSt relative to these other approaches, which we discuss briefly below.

The Microbiota Vault model advocates for the systematic “archiving” of microbiota across multiple domains—encompassing human, plant, animal, and ecosystem species—through the implementation of secure cryogenic storage, with the aim of addressing potential future restoration requirements in both human health and ecosystem conservation contexts (Dominguez Bello et al. 2025). This biobanking approach is principally concerned with the preservation of microbial specimens that have been diminished or eliminated as a consequence of industrialization, such as *Bifidobacterium longum* subsp. *infantis*. These efforts are clearly important and could be a part of stewardship efforts. However, MiSt is about actively intervening and changing existing practices that are causing harms, complementing the work of the Microbiota Vault, which focuses on preparing for future catastrophic failures. MiSt is about reducing changes to microbiomes that are associated with adverse health effects and maintaining environments that foster microbial resilience, rather than preserving snapshots of microbiomes in biobanks or vaults. Further, the vault approach focuses on preserving individual microorganisms (or microbial consortia), while MiSt focuses on microbial ecologies, treating microbiomes as collective phenomena inextricable from their habitats and contexts.

Another approach, the “conservation model” (Junker and Garwig 2025), is a species-centric model that prioritizes the prevention of species extinction, the maintenance of microbial diversity, and the protection of natural habitats. These efforts have culminated with the recent launch of the Microbial Conservation Specialist Group by the International Union for Conservation of Nature (Gilbert et al. 2025). The group will use microbial research to make suggestions for policy and action to restore deteriorated ecosystems, protect microbial biodiversity hotspots, and otherwise implement changes using microbiota to improve health. A microbiome stewardship approach is in line with conservation efforts but goes further by recognizing the complexity of the human social world, emphasizing that protecting microbial communities requires action beyond the field of conservation alone.

Proponents of the “rights-based approach” argue that while contemporary microbiome science and microbial ecology have moved beyond understanding microbes purely as pathogens—increasingly emphasizing their non-pathogenic and symbiotic roles in shaping ecosystems and the health of all life forms—these advances have yet to leave their mark on the international legal frameworks that govern human-microbial relations. To address this gap, Rizk and colleagues (2026) et al. (2026) propose two post-anthropocentric approaches to microbial rights: “Rights to Microbes” (RtM), which advocates for protecting microbial functions essential to the survival of ecosystems and multicellular living beings, and “Rights of Microbes” (RoM), which argues for recognizing the intrinsic and relational values of microbes themselves. Both frameworks represent distinct and nonetheless complementary pathways for elevating the legal and ethical status of microbial life in planetary governance.

MiSt is broadly compatible with the objectives of the Microbiota Vault, the conservation model, and the rights-based model; however, it offers the distinctive advantage of conceptualizing microbiomes simultaneously as essential ecological determinants of health for all life on Earth and as entities intrinsically intertwined with human existence. To support the enactment of MiSt across diverse domains, we have formulated guiding principles that are purposefully broad to be applicable across different contexts, not only with respect to efforts in the context of nature conservation. In addition, a stewardship approach does not conceive of conservation by removing the human impact, but rather by developing awareness of the entanglement of microbiomes with humans and other organisms, and the impact of human actions on these systems. Ultimately, we view MiSt as distinct from but complementary to other recently established approaches to safeguarding microbial life.

At present, One Health approaches at the microbial level are focused largely on controlling the emergence and spread of pathogenic microbes and antimicrobial resistance (Ginnan et al. 2025). Similarly, public health efforts directed towards microbes in many jurisdictions focus primarily on the control of pathogens. In contrast, MiSt focuses on the importance of microbiomes in supporting host health, as illustrated by ecosystem disruptions and consequent disease states that arise when microbiomes are altered by outside pressures (The Human Microbiome Project Consortium 2012, The Integrative HMP Research Network Consortium 2019, Lebeer et al. 2023). Its distinctive focus is thus on maintaining the microbial communities essential for host and ecosystem health. Nonetheless, we believe that MiSt is aligned with foundational principles of both One Health and public health approaches, and that they could benefit from incorporating MiSt into their frameworks.

Microbiome stewardship calls on us to recognize our interdependence with microbial ecosystems, and thus places responsibility with humans to act responsibly toward microbial ecosystems. Accepting microbiomes as a foundational element of our individual, and collective well-being and acting responsibly and with care, echoes the goals of broader ecosystem stewardship: to reduce or reverse the impact of preventable changes; adapt to changes that cannot be prevented; and shape future changes to enhance ecosystem resilience and host well-being (Chapin III et al. 2015).

## Data Availability

No new data were collected or used for the research reported in this article.
